# Acceptability of the dengue vaccination among parents in urban poor communities of Quezon City, Philippines before and after vaccine suspension

**DOI:** 10.1186/s13104-018-3766-y

**Published:** 2018-09-10

**Authors:** Ezra M. Valido, Ida Safitri Laksanawati, Adi Utarini

**Affiliations:** 1 Department of Public Health, Faculty of Medicine, Universitas Gadjah Mada, Yogyakarta, Indonesia; 2 Center for Topical Medicine, Faculty of Medicine, Universitas Gadjah Mada, Yogyakarta, Indonesia; 3 Pediatrics Department, Sardjito Hospital, Yogyakarta, Indonesia

**Keywords:** Acceptability, Dengue vaccine, Vaccine controversy, Philippines

## Abstract

**Objective:**

The study aims to illustrate the acceptability of the dengue vaccine before and after the dengue vaccination suspension in urban poor communities in Quezon City, Philippines.

**Results:**

There were 12 interviews conducted in November 2017 and 5 focus group discussions in January 2018, a month after vaccine program suspension with 41 participants. All participants were selected through purposive criterion sampling. Thematic analysis showed acceptability of the dengue vaccine was associated with parental experience with vaccination and dengue, trust in public health institutions and communication received by parents. Post-dengue vaccination suspension triangulation indicated that the parents regretted the experience, trust to public institutions was eroded and the communication strategy was deemed inadequate. This led to low vaccine acceptability post-vaccine suspension.

## Introduction

Dengue fever is a systemic viral illness that is transmitted through mosquitoes. The Philippines has a high burden of dengue and affects mostly children in urban areas [1]. In 2016, the Philippines initiated a mass dengue vaccination in three urban regions including Metro Manila. The initial vaccine coverage was low with low parental consent especially in Metro Manila. Initial vaccination targeted 4th graders aged 9 and above in public schools for 3 doses in a 6-month interval that were conducted by local health workers. Children with parental consent are the ones only eligible for vaccination. In December 2017, the dengue vaccination was suspended amid a vaccine controversy. The study aims to illustrate the acceptability of the dengue vaccine before and after the vaccine program suspension in an urban poor community.

## Main text

### Methods

Semi-structured in-depth interviews were conducted in November 2017 in urban poor communities of District 2 in Quezon City, Philippines. In January 2018, a month after dengue vaccine program suspension, 5 focus group discussions were conducted with parents of children who were vaccinated.

Parents of eligible children to the dengue vaccination were purposively selected. Two groups were recruited. Parents who consented and those who refused were interviewed. Economic income, educational attainment and sex were considered in selection. For focus group discussions, parents of children that were vaccinated were recruited. Participants were approached in communities. Participants were recruited from the 5 barangays of District 2, Quezon City.

The question guide for interview was developed using the determinants on vaccine hesitancy [2]. The results of the interviews were triangulated during the focus group discussions. Participants were informed about the research’s goals, those involve in the research and the funding before the interviews and focus group discussions. Paraphrasing and summarizing the participant’s responses with feedback was asked for every question to ensure that the views and perspectives of the participants are noted. Additional questions explored the responses provided by participants and clarify issues raised.

The interviews were conducted in the interviewee’s home and focus group discussions in community centers. Only the research team and participants were present. All interviews lasted < 30 min and 40 min for focus group discussions. The interviews and focus group discussions were conducted and supervised by the primary author with assistance from a trained research assistant. The primary author is a male medical doctor who worked in primary care with training in management and in training for implementation research. The collection was in the local language.

The interviews and focus group discussions were audio recorded and transcribed verbatim with notes on non-verbal cues. No transcripts were returned to participants. Transcripts were analyzed thematically with three coders. All coders were doctors with degrees in management. Pre-determined codes were used as guide. There were minimal differences in codes and consensus were sought for variations. There was no repeat interview conducted nor repeat recruitment. For the focus group discussions, three coders read and independently constructed a code list. Coders were oriented on thematic results of the interviews prior to coding. The categories were compared and crosschecked with themes from the interviews. Emerging themes were noted. Consensus were sought for variations. Codes were managed using the OpenCode 4.02 [3].

### Results

There were 12 interviews. There were 6 females and 6 males, 8 were non-indigents and 4 were indigents, 4 had college degrees, 4 with high school degrees and 4 had no formal education. For the 5 focus group discussions there were 41 total participants. Except for one, the rest were female participants. The results are presented thematically below in Table 1.

**Table 1:** Key themes in the interviews and focus group discussions

Key themes	Key pre-suspension categories	Illustrative quotes (interviews)	Key post-suspension categories	Illustrative quotes (focus group discussion)
Parental experience in dealing with vaccination and dengue	Previous vaccine experience Severity of dengue The vaccine was free	“*I support vaccination*…*I am a senior citizen and I have my anti*-*flu and anti*-*pneumonia vaccinations*” (non-indigent, without college degree, male) “…*I am afraid experiencing severe dengue again*” (non-indigent, without college degree, female, consented) “*I wanted the vaccine because it was free*” (non-indigent, without college degree, male, consented)	Fear for the child Regret and guilt Hypervigilance to symptoms	“*We were afraid they could die because of the vaccination*” “*I felt guilty because my child did not want to be vaccinated*… *but I insisted*” “*Now, when my child complains of a symptom*…*I would ask myself, is this the effect of Dengvaxia?*”
Parental trust on the public health institutions	Trust the public health institutions Health workers recommendation	“*The vaccines came from DOH. Government will not do a program without good intentions for it*” (non-indigent, without college degree, female, consented) “…*they (health workers) were supportive of the vaccination.*” (Indigent, female)	Corruption was involved Lack of trustworthy information sources Lost trust in government health programs	“… *it was obvious that there were large sums of money involved*” “*We have not heard anything from the health center about the vaccine*…” “*Now I doubt the health department’s programs…before if from DOH I will take it. Now, no*”
Communication received by parents	Negative vaccine messages Vaccination happened suddenly Community-based interventions	“…*on the news someone died from the dengue vaccine*…*I got scared and not consented*” (Non-indigent, with college degree, female, did not consent) “*The vaccination happened suddenly*” (indigent, female, did not consent) “*Information should come first from DOH then disseminated to the barangays and health centers.*” (Non-indigent, without college degree, male, consented)	Poor vaccine coherence Community-based interventions Assurance of child safety	“…*if the child had been injected with Dengvaxia and had no dengue, they would develop severe dengue and severe dengue cause death*” “*It (community meetings) is better if there are questions and could be answered. Like an open forum*…” “…*will children develop severe disease when vaccinated*… *what is the effect? Is it because of the dengue vaccine?*”

#### Parental experience with the disease and vaccination

The acceptability of the dengue vaccine was rooted in the previous vaccine experience of parents. Parents understanding of the dengue vaccine was limited because it was new, and they believed the dengue vaccine was like other government vaccines. Parents with pleasant experience with government vaccinations have accepted the vaccine. Some parents refused the vaccination because their children had an adverse reaction or are afraid of the injection. Likewise, parents who refused the vaccination demanded more information on vaccine safety and benefits especially if they have previous reactions from other vaccines. Those who refuse tend to know more about vaccines and refuse the dengue vaccine only among other vaccines. They believe the vaccine has not been proven effective because it was new. Moreover, the vaccine was provided free by government and was seen to improve access. Parents’ experience on hospital and private vaccinations is expensive.

The perceived severity of dengue, likewise, influences the vaccine acceptability. Parents whose children who had severe forms of dengue or knows someone who had died or has been critically hospitalized are likely to accept the vaccine while those whose children had mild dengue were less likely to accept.

During the controversy, most vaccine messages were on vaccine related deaths. This has caused stress and anxiety among parents. They were fearful for their child’s health. Parents were hypervigilant on their children’s health and changed their health seeking behavior. They preferred private health practitioners. Parents suffered both from an emotional turmoil and economic loss. The vaccination experience is generally regretted. Interestingly, anger towards government though is not dominantly seen as the parents rationalize that they consented to the vaccination.

#### Trust in public health institutions

Parents express that they have limited knowledge on what the dengue vaccine is and what it does, but they trust the vaccinators, the vaccination program and the health institutions that led them to accepting the vaccine. Parents has been influenced by the recommendations of health workers in the dengue vaccinations.

During the vaccine controversy, the trust in public health institutions has been eroded especially that the vaccination program was linked with corruption practices by health leaders. Trust in information being received from these institutions has been low. Parents find it difficult to find trustworthy information sources. Health workers, especially local health workers and medical doctors, are the most cited trustworthy information sources but the lack of avenues and confidence in discussing what happened to the vaccination has led to loss of trust in the dengue vaccination but also to other school vaccinations and public health programs.

#### Communication received by parents

The initial implementation of the vaccination happened suddenly, and some parents wanted to participate but unable to. This suddenness in implementation is seen to be contributory to the general lack of knowledge on vaccine specific details such as number of doses, intervals, risks and vaccination site. Parents preferred community-based interventions in communication with their local health workers in multiple forms even if vaccination sites are in schools. They needed more information on dengue vaccine benefits, risks and safety.

With the vaccine controversy, the communication received was perceived to be inadequate. Most of the parents received information from the news and not from their local health workers. Dengue vaccine coherence and perceived effectiveness were low. Some parents associate having the dengue vaccine as a risk to severe dengue regardless of previous history. Severe dengue is equated to death. Some parents think the dengue vaccine is a poison and they need an antidote. Others thinks that the vaccine increases the risks of their children to other severe diseases. Information need by parents centered on child safety. Community-based communication strategies is preferred to allow parents to ask questions with the local medical doctors in their communities are expected to conduct the information dissemination.

### Discussion

The Philippine dengue vaccination is the only mass dengue vaccination to date with more than 800,000 individuals vaccinated. Vaccination controversy due to perceived increased risk of serious dengue illness and death with the media associating the vaccine program to politics and corruption has caused low vaccine acceptability. In the study, dengue vaccine acceptability is linked to the parental experience in dealing with vaccination in general and dengue, trust in public health institutions, and the communication received by parents.

The dengue vaccine is new, like other new vaccines suffer from perception of lack of testing and efficacy [4], and vaccine specific characteristics are different from routine vaccines used in other mass vaccination. Parents rely on their own experience from their previous vaccination that includes their personal and familial encounter with vaccines, their experience with the disease and their communities’ experience. Consistent with other studies [5–10], those who refused the vaccine demanded more information on vaccine safety emanating from an experience of an adverse reaction. Likewise, parent–child interaction for decision making is observed, like in other vaccinations [11, 12], involving older children especially in considering the child’s apprehension, discomfort and previous adverse reaction. This was apparent especially among those who refused. The parents who accepted the dengue vaccine believe that children are not yet capable to decide for their health and insisted on the vaccination. The perceived severity of the disease is likewise factored in during decision making especially when death in the family or community is experienced. Like for other vaccines the more severe the disease it prevents the better the acceptability [4, 13, 14]. For dengue the difference in perception observed is due to the range of disease presentation.

Parents’ trust to health institutions is key in the acceptability of the dengue vaccine. The source of the vaccination and credibility of the institutions matter more than the information received [4]. The health workers’ confidence in discussing the vaccine and vaccination process improves acceptability. The dengue vaccine is new, and vaccine specific characteristics are different from routine vaccines used in other mass vaccination. The immediate initial implementation of the vaccination and lack of socialization explains the inadequacy of knowledge and confidence among local health workers in discussing the vaccine. The lack of socialization makes negative vaccine messages more potent because the expected individuals to defend the vaccine and vaccination are unable to adequately answer parental concerns. This makes parents hesitant.

The acceptability changes throughout implementation of a program [15]. The dengue vaccine controversy triggered a change of attitude and understanding of parents who previously accepted the vaccine. The negative vaccine news generated fear for the vaccine, regret and guilt in participation and mistrust on the vaccination program. Repeated focus on presumed vaccine deaths on mass media eroded trust to the dengue vaccine. Moreover, the association of the dengue vaccination to corruption of public health officials and politicians decreased the trust on the vaccination. The parents have increased demand for specific information with regards to vaccine safety. However, information from local health centers and from the health department has been perceived to be inadequate and non-reassuring. Health workers are the primary sought after information sources [6, 16–21] and community-based communications are preferred. The lack of trust in the dengue vaccine, the vaccinators and those handling the vaccination program made parents confident in deciding to refuse completion of vaccine dose, participate in other school vaccination and other school health programs.

The parental experience with vaccination and dengue, trust in public health institutions, and the communication received by parents are inter-related. Communication strategies play vital role in addressing parental concerns and can be tailor-fitted and targeted. The lack of proper and adequate communication led to decrease trust to providers and eventually led to poor experience. Poor experience is a justification for non-acceptance of vaccines. During vaccine controversies, there is a demand for more and specific information. The engagement with parents in eliciting information need needs to be integrated in communication plans. Local health workers must have the capacity to respond to the need. Communication strategies must not be limited to knowledge transfers but rather focus on building trust.

## Limitations

The study is an exploratory qualitative research. Concerns related to the dengue vaccine may vary from other new and available vaccines. The controversy surrounding the dengue vaccine both in content and coverage is dissimilar from other vaccine controversies. The dengue vaccination in the study has been used in mass vaccination in schools and at the local health centers and differs from routine vaccination experienced in other localities.
